# Global Fast Terminal Fuzzy Sliding Mode Control of Quadrotor UAV Based on RBF Neural Network

**DOI:** 10.3390/s25041060

**Published:** 2025-02-10

**Authors:** Weidong Chen, Yuanchun Ding, Falu Weng, Chuanfu Liang, Jiawei Li

**Affiliations:** 1 School of Electrical Engineering and Automation, Jiangxi University of Science and Technology, Ganzhou 341000, China; 6720220734@mail.jxust.edu.cn (W.C.); 6720220727@mail.jxust.edu.cn (C.L.); 6720220723@mail.jxust.edu.cn (J.L.); 2 Ganzhou Key Laboratory of Industrial Safety and Emergency Technology, Jiangxi Provincial Key Laboratory of Safe and Efficient Mining of Rare Metal Resource, Jiangxi University of Science and Technology, Ganzhou 341000, China

**Keywords:** unmanned aerial vehicle, global fast terminal sliding mode control, RBF neural network, fuzzy control, adaptive control

## Abstract

In this paper, a global fast terminal fuzzy sliding mode control scheme based on the radial basis function (RBF) neural network is proposed for quadrotor unmanned aerial vehicle systems in the presence of external disturbances, system model uncertainty, and time-varying mass. Firstly, the dynamic model of the quadrotor is divided into two subsystems, i.e., an outer-loop control subsystem and an inner-loop control subsystem. Secondly, an adaptive sliding mode controller is used to control the outer-loop control subsystem, which includes the adaptive laws estimating the time-varying mass and external disturbances. In the inner-loop control subsystem, a global fast terminal fuzzy sliding mode controller, which is based on the RBF neural network, is designed to control the attitude of a quadrotor. In this method, the system model uncertainty is approximated using the RBF neural network. Simultaneously, an adaptive fuzzy controller is introduced to estimate the switching gain and eliminate external disturbances, and the chattering phenomenon is eliminated effectively. Finally, simulations are provided to demonstrate the effectiveness of the proposed control scheme.

## 1. Introduction

Quadrotor unmanned aerial vehicles (QUAVs) have the advantages of simple structure, low cost, vertical takeoff, stable hovering, etc., so they are widely used in civil and military fields [1]. In the civil field, QUAVs are used in ground monitoring, plant protection in agriculture and forestry, and to combat the COVID-19 pandemic [2]. However, in certain circumstances, QUAVs may experience mass changes during flight. For example, when using QUAVs to spray fungicide and distribute relief food in public and open places during the period of fighting the COVID-19 pandemic, the total mass of the QUAVs was time-varying [3] or changed abruptly at a certain moment. Thus, it is crucial to consider the mass change for controlling the QUAVs. Moreover, QUAVs are under-actuated, strongly coupled, and nonlinear systems. They are susceptible to external disturbances [4] and system model uncertainty. Therefore, stability analysis and controller design for QUAVs are still challenging.

Fortunately, to address these issues, many efforts have been made by scholars in recent years, and some achievements have been accomplished. As a result, a variety of nonlinear robust control methods have been designed, for example, the sliding mode control (SMC) [5], backstepping control [6], active disturbance rejection control (ADRC) [7], adaptive SMC [8], terminal sliding mode control [9], neural network control [10], etc.

SMC has the advantages of robustness, ease of implementation, and fast response speed and is widely used in various control systems [11]. In [12], a sliding mode saturation control method was proposed for the altitude and attitude control problem of a QUAV, and the simulation results show that the sliding mode controller has good performance and robustness to disturbances. However, the main drawback of the SMC is chattering phenomena and slow convergence [13]. Fast terminal sliding mode control (FTSMC) improves the convergence speed of the system by introducing an exponential term sliding mode surface [14] so that the system converges in a finite time. In [15], the authors proposed a global fast terminal sliding mode control (GFTSMC) method to control a QUAV, and simulation results verify its superior performance. In [16], the authors proposed an adaptive sliding mode control (ASMC) method for QUAVs that takes into account input saturation, and simulations were conducted to verify the effectiveness of the proposed control method. However, the proposed method (ASMC) does not consider the anti-chattering phenomenon. In [17], a new adaptive law was proposed, and it achieves fast adaptation and significant chattering reduction. However, the time-varying mass is not taken into account in all these controllers. This limits the adaptability and anti-chattering phenomenon of the controllers. This paper proposed a control method that is more appropriate for engineering applications due to its strong anti-chattering, strong anti-disturbance, fast convergence speed, and great robustness to system model uncertainty.

Integrated control combining fuzzy control, SMC, and neural network control has become a new research hotspot [18,19,20]. The radial basis function (RBF) neural network was proposed in 1988. Research on RBF networks has shown that RBF neural networks can approximate any nonlinear function with a compact set and arbitrary accuracy [21]. In [22], the authors provided a PID control system for QUAV trajectory tracking control that is based on fuzzy control and RBF neural networks. The simulation results demonstrate that the scheme may greatly increase the accuracy and robustness of QUAV trajectory tracking control. The literature shows that the combination of neural network control and PID control can make the controlled system more efficient and stable. However, the QUAV system is essentially nonlinear, and this compound control method has no significant effect on improving the control system’s ability to deal with uncertain changes. In [23], the authors proposed an improved novel RBF neural network controller. Simulation results show that this method significantly accelerates the system convergence speed and effectively improves the control accuracy. However, the controller only utilizes a single neural network control method with poor overall robustness.

Fuzzy control has the characteristics of not requiring a mathematical model of the controlled object and strong robustness. Fuzzy rules are used to efficiently estimate the sliding mode switching gain, which in turn removes the external disturbance term from the system and eliminates chattering [24]. In [25], an adaptive fuzzy sliding mode control scheme was designed for the uncertainty and external disturbance problems in underdriven mechanical systems, and experiments show that the method has good trajectory tracking performance. The control method uses switching variables to reduce fuzzy rules and computation, but this affects how practical the control method is, and it does not completely solve the chattering problem. In [26], the authors proposed a genetic algorithm-improved fuzzy sliding mode control method to control the position and altitude trajectory of a QUAV, and the simulation results show that the method can not only significantly solve the chattering problem existing in the traditional model (SMC), but also further improve the flying performance of the QUAV. However, the computational complexity of this method is high, and in scenes with high real-time requirements, it may not be able to process the information in time, affecting the control effect. On the other hand, the fuzzy rules are randomly generated and optimized using the genetic algorithm, which avoids relying on expert information but may lead to a lack of reasonableness of the rules.

Summarizing the advantages and disadvantages of the control methods described in the above literature, this paper aims to study QUAVs with external disturbances, system model uncertainty, and time-varying mass, which are currently less studied. It remains necessary and meaningful to achieve some results in this field. This is the main motivation for this paper’s research. In this paper, fuzzy control, the radial basis function (RBF) neural network, and sliding mode control are combined. On this basis, a global fast terminal fuzzy sliding mode control scheme based on the radial basis function (RBF) neural network is proposed for QUAV systems in the presence of external disturbances, system model uncertainty, and time-varying mass. Firstly, the dynamic model of the quadrotor is divided into two subsystems, i.e., an outer-loop control subsystem and an inner-loop control subsystem. Secondly, the time-varying mass and unknown disturbances are estimated using the adaptive laws designed in the ASMC controller, which is used to control the position subsystem. In the inner-loop control subsystem, a global fast terminal sliding mode controller, which is based on the RBF neural network (GFTFSMC-RBF), was designed to control the attitude of a quadrotor. In this method, the system model uncertainty is approximated using the RBF neural network. Simultaneously, an adaptive fuzzy controller is introduced to estimate the switching gain. The switching gain is used to eliminate the external disturbances, and the chattering phenomenon is eliminated effectively. The main contributions of this study include the following:

(i) The proposed control method combines global fast terminal sliding mode control, the RBF neural network, and fuzzy control, which have the advantages of fast finite time convergence, high anti-chattering, high anti-disturbance, and great robustness to system uncertainty;
(ii) The adaptive laws are formulated to estimate the upper bounds of time-varying mass and external disturbances appearing in the position subsystems;
(iii) A global fast terminal fuzzy sliding mode controller, which is based on the RBF neural network, is designed to control the attitude of a quadrotor. The approximation of system model uncertainty using the RBF neural network to improve the anti-disturbance capability of the control system is determined. An adaptive fuzzy controller is used to estimate the switching gain and eliminate external disturbances, effectively reducing chattering;
(iv) Replacing the traditional sign function (sign(s)) with an improved saturation function further contributes to the chattering attenuation.

The rest of this paper is organized as follows: In Section 2, the dynamics model of a quadrotor UAV is described. Section 3 designs the ASMC and the proposed GFTFSMC-RBF controllers to control the position and attitude of the QUAV. Section 4 demonstrates the effectiveness of the proposed controller by discussing and analyzing the simulation results. Section 5 provides the conclusion of this paper.

## 2. System Modeling and Problem Description

**Figure 1:**
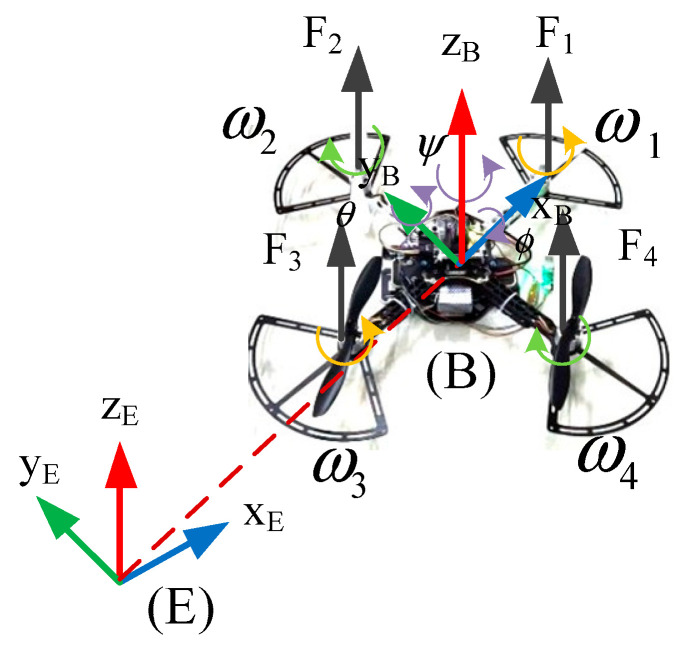
QUAV’s configuration.

The earth frame (E), which is attached to the center of the earth, and the body frame (B), which is fixed to the QUAVs, are established initially to establish the QUAV’s mathematical model. According to Figure 1, the quadrotor UAV’s Euler angles are represented by the roll (ϕ), pitch (θ), and yaw (ψ), and $F_{1}$, $F_{2}$, $F_{3}$, and $F_{4}$ are the four-rotor elevations of the QUAVs, respectively [27].

To facilitate the design of the QUAVs controller, the following assumptions are made [28]:

**Hypothesis** **1.** *A QUAV is a rigid body with uniformly distributed mass and center symmetry.*

**Hypothesis** **2.** *The mass and inertia of a QUAV are constant.*

**Hypothesis** **3.** *Thrust and drag on each wing of a QUAV are proportional to the square of propeller speed.*

$R_{b}^{e}$ is the homogenous matrix transformation [29].

(1) $$R_{b}^{e}=\left[\begin{array}{ccc} C\theta C\psi & C\psi S\theta S\phi -S\psi C\theta & C\psi S\theta C\phi +S\psi S\phi \\ C\theta S\psi & S\psi S\theta S\phi +C\psi C\phi & S\psi S\theta C\phi -S\psi C\phi \\ -S\theta & S\phi C\theta & C\phi C\theta \end{array}\right]$$

where Cθ means cosθ, Sθ means sinθ, and so forth. The dynamics of the quadrotor UAV can be modeled using the Newton–Euler formulation as follows:

(2) $$\left\{\begin{array}{l} \dot{\Lambda }=\nu \\ m\ddot{\Lambda }=F_{f}-F_{g}-F_{t}\\ \dot{\Phi }=W\omega_{b}\\ J{\dot{\omega }}_{b}=-\omega_{b}\times (J\omega_{b})+\Gamma_{f} \end{array}\right.$$

Λ is the position of the QUAV’s center of mass relative to the earth frame (E); m is the total mass of the structure, and J is a symmetric positive definite constant inertia matrix of the QUAVs for $\omega_{b}$.

(3) $$J=\left[\begin{array}{ccc} I_{x} & 0 & 0\\ 0 & I_{y} & 0\\ 0 & 0 & I_{Z} \end{array}\right]$$

$F_{f}$ is the resultant of the forces generated by the four rotors.

(4) $$F_{f}=\left[\begin{array}{c} \cos\phi \cos\psi \sin\theta +\sin\psi \sin\phi \\ \cos\phi \sin\theta \sin\psi -\sin\phi \cos\psi \\ \cos\phi \cos\theta \end{array}\right]\sum_{i=1}^{4}F_{i}$$

(5) $$F_{i}=K_{p}\omega_{i}^{2}$$

where $K_{p}$ is the lift coefficient and $\omega_{i}$ is the rotational speed of the motor. The gravity force $F_{g}$ is given by

(6) $$F_{g}={\left[0\;0\;mg\right]}^{T}$$

where Φ is the Euler angles of the QUAVs for the earth frame (E) and $\omega_{b}$ is the angular velocity of the airframe for the body frame (B). W denotes the transformation matrix between $\dot{\Phi }$ and $\omega_{b}$.

(7) $$\left\{\begin{array}{c} \Phi ={\left[\phi \;\theta \;\psi \right]}^{T},\omega_{b}={\left[\omega_{x}\;\omega_{y}\;\omega_{z}\right]}^{T}\\ W=\left[\begin{array}{ccc} 1 & \tan\theta \sin\phi & \tan\theta \cos\phi \\ 0 & \cos\phi & -\sin\phi \\ 0 & \sin\phi /\cos\theta & \cos\phi /\cos\theta \end{array}\right]\\ \dot{\Phi }=W\omega_{b} \end{array}\right.$$

$F_{t}$ is the resultant of the drag forces along the ($x_{b},y_{b},z_{b}$) axis.

(8) $$F_{t}=\left[\begin{array}{ccc} K_{ftx} & 0 & 0\\ 0 & K_{fty} & 0\\ 0 & 0 & K_{ftz} \end{array}\right]\dot{\Lambda }$$

where $K_{ftx}$, $K_{fty}$, and $K_{ftz}$ are the coefficients of drag in translation. $\Gamma_{f}$ is the moment developed by the quadrotor according to the body’s fixed frame. It is expressed as follows [30]:

(9) $$\Gamma_{f}=\left[\begin{array}{l} \tau_{x}\\ \tau_{y}\\ \tau_{z} \end{array}\right]=\left[\begin{array}{l} \frac{\sqrt{2}}{2}d(\omega_{1}^{2}-\omega_{2}^{2}-\omega_{3}^{2}+\omega_{4}^{2})\\ \frac{\sqrt{2}}{2}d(\omega_{1}^{2}+\omega_{2}^{2}-\omega_{3}^{2}-\omega_{4}^{2})\\ K_{f}(\omega_{1}^{2}-\omega_{2}^{2}+\omega_{3}^{2}-\omega_{4}^{2}) \end{array}\right]$$

where d is the distance between the center of the quadcopter and the center of a propeller and $K_{f}$ is the drag coefficient. In summary, the complete model of the dynamics of a QUAV can be represented as follows [31]:

(10) $$\left\{\begin{array}{l} \ddot{x}=\left\{\left(\cos\phi \sin\theta \cos\psi +\sin\phi \sin\psi \right)U_{1}-K_{ftx}\dot{x}\right\}\frac{1}{m}+\Delta_{x}\\ \ddot{y}=\left\{\left(\cos\phi \sin\theta \sin\psi -\sin\phi \cos\psi \right)U_{1}-K_{fty}\dot{y}\right\}\frac{1}{m}+\Delta_{y}\\ \ddot{z}=\left\{\left(\cos\phi \cos\theta \right)U_{1}-K_{ftz}\dot{z}\right\}\frac{1}{m}-g+\Delta_{z}\\ \ddot{\phi }=\frac{\left(I_{y}-I_{z}\right)}{I_{x}}\dot{\theta }\dot{\psi }+\frac{dU_{2}}{I_{x}}+\Delta_{\phi }\\ \ddot{\theta }=\frac{\left(I_{z}-I_{x}\right)}{I_{y}}\dot{\phi }\dot{\psi }+\frac{dU_{3}}{I_{y}}+\Delta_{\theta }\\ \ddot{\psi }=\frac{\left(I_{x}-I_{y}\right)}{I_{z}}\dot{\theta }\dot{\phi }+\frac{U_{4}}{I_{z}}+\Delta_{\psi } \end{array}\right.$$

Here, $\Delta_{i}(i=x,y,z,\phi ,\theta ,\psi )$ denotes system uncertainty and external disturbances. $U_{1}$, $U_{2}$, $U_{3}$, and $U_{4}$ are the control inputs of the system, which are written according to the angular velocities of the four propellers as follows:

(11) $$\left[\begin{array}{l} U_{1}\\ U_{2}\\ U_{3}\\ U_{4} \end{array}\right]=\left[\begin{array}{cccc} K_{p} & K_{p} & K_{p} & K_{p}\\ \frac{\sqrt{2}d}{2I_{x}}K_{p} & -\frac{\sqrt{2}d}{2I_{x}}K_{p} & -\frac{\sqrt{2}d}{2I_{x}}K_{p} & \frac{\sqrt{2}d}{2I_{x}}K_{p}\\ \frac{\sqrt{2}d}{2I_{y}}K_{p} & \frac{\sqrt{2}d}{2I_{y}}K_{p} & -\frac{\sqrt{2}d}{2I_{y}}K_{p} & -\frac{\sqrt{2}d}{2I_{y}}K_{p}\\ \frac{1}{I_{z}}K_{f} & -\frac{1}{I_{z}}K_{f} & \frac{1}{I_{z}}K_{f} & -\frac{1}{I_{z}}K_{f} \end{array}\right]\left[\begin{array}{l} \omega_{1}\\ \omega_{2}\\ \omega_{3}\\ \omega_{4} \end{array}\right]$$

## 3. The Control Strategy

System Equation (10) is rewritten in state space for controller design. The model of the state space adopted by the control system is as follows:

(12) $$\dot{X}=G(X,U)+\Delta_{i}(i=x,y,z,\phi ,\theta ,\psi )$$

where X is the state vector and U is the control input vector. The state vector is selected as

(13) $${X=[x}_{1}{,x}_{2},\cdots {,x}_{12}{]}^{T}={\left[x,\dot{x},y,\dot{y},z,\dot{z},\phi ,\dot{\phi },\theta ,\dot{\theta },\psi ,\dot{\psi }\right]}^{T}$$

Combining Equations (10)–(13) gives

(14) $$G(X,U)=\left\{\begin{array}{c} {\dot{x}}_{1}=x_{2}\\ {\dot{x}}_{2}=a_{1}x_{2}+\frac{U_{x}}{m}+\Delta_{x}\\ {\dot{x}}_{3}=x_{4}\\ {\dot{x}}_{4}=a_{2}x_{4}+\frac{U_{y}}{m}+\Delta_{y}\\ {\dot{x}}_{5}=x_{6}\\ {\dot{x}}_{6}=a_{3}x_{6}+\frac{U_{z}}{m}-g+\Delta_{z}\\ {\dot{x}}_{7}=x_{8}\\ {\dot{x}}_{8}=a_{4}x_{10}x_{12}+b_{1}U_{2}+\Delta_{\phi }\\ {\dot{x}}_{9}=x_{10}\\ {\dot{x}}_{10}=a_{5}x_{8}x_{12}+b_{2}U_{3}+\Delta_{\theta }\\ {\dot{x}}_{11}=x_{12}\\ {\dot{x}}_{12}=a_{6}x_{8}x_{10}+b_{3}U_{4}+\Delta_{\psi } \end{array}\right.$$

(15) $$\left\{\begin{array}{c} a_{1}=-\frac{K_{ftx}}{m},a_{2}=-\frac{K_{fty}}{m},a_{3}=-\frac{K_{ftz}}{m}\\ a_{4}=\frac{\left(I_{y}-I_{z}\right)}{I_{x}},a_{5}=\frac{\left(I_{z}-I_{x}\right)}{I_{y}},a_{6}=\frac{\left(I_{x}-I_{y}\right)}{I_{z}}\\ b_{1}=\frac{d}{I_{x}},b_{2}=\frac{d}{I_{y}},b_{3}=\frac{1}{I_{z}}\\ U_{x}=\left(\cos\phi \sin\theta \cos\psi +\sin\phi \sin\psi \right)U_{1}\\ U_{y}=\left(\cos\phi \sin\theta \sin\psi -\sin\phi \cos\psi \right)U_{1}\\ U_{z}=\left(\cos\phi \cos\theta \right)U_{1} \end{array}\right.$$

From Equations (10) and (15), $U_{1}$, $\phi_{d}$, and $\theta_{d}$ can be found [32]:

(16) $$\left\{\begin{array}{l} U_{1}=\frac{U_{z}}{\cos\theta_{d}\cos\phi_{d}}\\ \phi_{d}=\arcsin\left(\frac{U_{y}\cos\psi -U_{x}\sin\psi }{U_{z}}\right)\\ \theta_{d}=\arctan\left(\frac{U_{y}\cos\psi +U_{x}\sin\psi }{U_{z}}\right) \end{array}\right.$$

**Figure 2:**
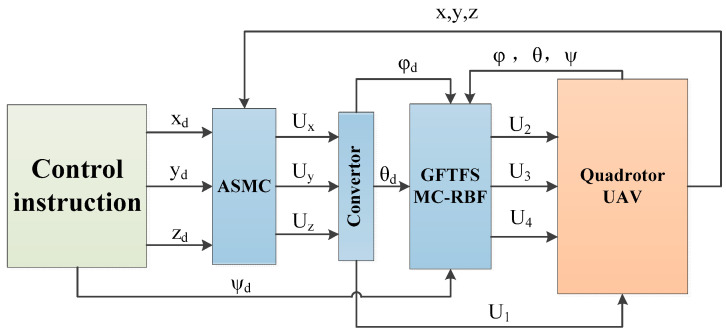
Control scheme.

From Equation (2), the QUAV’s system has 6 outputs and only 4 inputs, which is a typical underdriven system. The rotational motions do not rely on translational motion while the opposite is not true. The QUAV’s attitude and position are therefore managed via a double-loop control system. ASMC is used for position control in the outer loop, while GFTFSMC-RBF is used for attitude control in the inner loop. In Figure 2, the control scheme is displayed. The control instruction first specifies a desired yaw angle $\psi_{d}$ and three desired trajectories, $x_{d}$, $y_{d}$, and $z_{d}$. $U_{x}$, $U_{y}$, and $U_{z}$ are then derived from the ASMC control in the position direction. $U_{x}$, $U_{y}$, and $U_{z}$ are then replaced into Equation (16) to produce $U_{1}$, $\phi_{d}$, and $\theta_{d}$. $\phi_{d}$ and $\theta_{d}$ are then substituted into the GFTFSMC-RBF attitude controller to produce $U_{2}$, $U_{3}$, and $U_{4}$. Finally, the outputs of the three actual positions and three attitudes of the QUAVs are used as feedback for ASMC and GFTFSMC-RBF to realize the trajectory tracking control of the QUAVs.

### 3.1. Position Controller Design

From Equation (14), the state space expression of the position subsystem is given by

(17) $$\left\{\begin{array}{c} {\dot{x}}_{1}=x_{2}\\ {\dot{x}}_{2}=a_{1}x_{2}+\frac{U_{x}}{m}+\Delta_{x}\\ {\dot{x}}_{3}=x_{4}\\ {\dot{x}}_{4}=a_{2}x_{4}+\frac{U_{y}}{m}+\Delta_{y}\\ {\dot{x}}_{5}=x_{6}\\ {\dot{x}}_{6}=a_{3}x_{6}+\frac{U_{z}}{m}-g+\Delta_{z} \end{array}\right.$$

To make the subsequent analysis concise and readable, some of the involved disturbance components in Equation (17) are rescaled as ${\overset{⌣}{\Delta }}_{z}=\Delta_{z}≜\frac{\Delta_{z}}{m}$, ${\overset{⌣}{\Delta }}_{x}=\Delta_{x}≜\frac{\Delta_{x}}{m}$, and ${\overset{⌣}{\Delta }}_{y}=\Delta_{y}≜\frac{\Delta_{y}}{m}$.

**Hypothesis** **4.** *The assumption is that the uncertainty and external disturbances of the system are bounded, and its derivatives are also bounded.* $\left|{\overset{⌣}{\Delta }}_{x}\right|\leq \tau_{x}$*,* $\left|{\overset{⌣}{\Delta }}_{y}\right|\leq \tau_{y}$*,* $\left|{\overset{⌣}{\Delta }}_{z}\right|\leq \tau_{z}$*,* $\left|\Delta_{\phi }\right|\leq \tau_{\phi }$*,* $\left|\Delta_{\theta }\right|\leq \tau_{\theta }$*,* $\left|\Delta_{\psi }\right|\leq \tau_{\psi }$*,* $\left|{\dot{\overset{⌣}{\Delta }}}_{x}\right|\leq \tau_{dx}$*,* $\left|{\dot{\overset{⌣}{\Delta }}}_{y}\right|\leq \tau_{dy}$*,* $\left|{\dot{\overset{⌣}{\Delta }}}_{z}\right|\leq \tau_{dz}$*,* $\left|{\dot{\Delta }}_{\phi }\right|\leq \tau_{d\phi }$*,* $\left|{\dot{\Delta }}_{\theta }\right|\leq \tau_{d\theta }$*, and* $\left|{\dot{\Delta }}_{\psi }\right|\leq \tau_{d\psi }$ *as well as all of* $\tau_{x}$*,* $\tau_{y}$*,* $\tau_{z}$*,* $\tau_{\phi }$*,* $\tau_{\theta }$*,* $\tau_{dx}$*,* $\tau_{dy}$*,* $\tau_{dz}$*,* $\tau_{d\phi }$*,* $\tau_{d\theta }$*, and*$\tau_{d\psi }$*are an unknown positive constant*.

**Hypothesis** **5.** *Assuming that the rate of change in the external disturbance is relatively slow for a sufficiently long time* $t_{0}$, $\lim_{t\to t_{0}}{\dot{\Delta }}_{i}=0$.

We use the *z* channel as an example to design its controller and define tracking error as

(18) $$e_{z}=z-z_{d}$$

We construct the sliding surface as

(19) $$s_{z}=k_{z}e_{z}+{\dot{e}}_{z}$$

Taking the derivative of (19), we can obtain

(20) $${\dot{s}}_{z}=k_{z}{\dot{e}}_{z}+{\ddot{e}}_{z}$$

Equation (20) combined with Equations (17)–(19) gives

(21) $$\begin{array}{ll} {\dot{s}}_{z} & =k_{z}{\ddot{e}}_{z}+\ddot{z}-{\ddot{z}}_{d}\\ & =k_{z}{\ddot{e}}_{z}+\frac{1}{m}\left(\cos\varphi \cos\theta \right)U_{1}-\frac{1}{m}K_{ftz}\dot{z}-g+\frac{\Delta_{z}}{m}-{\ddot{z}}_{d}\\ & =k_{z}{\ddot{e}}_{z}+\frac{U_{v}+\Delta_{z}}{m}-g-{\ddot{z}}_{d} \end{array}$$

where $U_{v}=(\cos\phi \cos\theta )U_{1}-K_{ftz}\dot{z}$ and $\Delta_{z}$ are the design’s virtual control inputs and external disturbances. $U_{v}$ is designed as follows:

(22) $$\left\{\begin{array}{l} U_{v}=\hat{m}{\bar{U}}_{v}-{\hat{\Delta }}_{z}\\ {\bar{U}}_{v}=g+{\ddot{z}}_{d}-k_{z}{\dot{e}}_{z}-c_{1}s_{z} \end{array}\right.$$

where $c_{1}>0$. $\hat{m}$ and ${\hat{\Delta }}_{z}$ represent the estimated total mass and total disturbance of the quadrotor UAV, respectively. Substituting Equation (22) in Equation (21) gives

(23) $${\dot{s}}_{z}=k_{z}{\ddot{e}}_{z}+\frac{\hat{m}{\bar{U}}_{v}-{\hat{\Delta }}_{z}+\Delta_{z}}{m}-g-{\ddot{z}}_{d}$$

We define ${\tilde{\Delta }}_{z}=\Delta_{z}-{\hat{\Delta }}_{z}$ and $\tilde{m}=m-\hat{m}$, which are the mass and total disturbance estimate errors, respectively. According to Hypothesis 2, ${\dot{\tilde{\Delta }}}_{z}=-{\dot{\hat{\Delta }}}_{z}$ and $\dot{\tilde{m}}=-\dot{\hat{m}}$.

**Proof.**  The Lyapunov candidate functions are designed as follows:

(24) $$V=\frac{1}{2}s_{z}^{T}s_{z}+\frac{1}{2m\eta }{\tilde{\Delta }}_{z}^{T}{\tilde{\Delta }}_{z}+\frac{1}{2m\sigma }{\tilde{m}}^{2}$$

The derivative of the Lyapunov candidate functions is obtained:

(25) $$\dot{V}=s_{z}^{T}{\dot{s}}_{z}+\frac{1}{2m\eta }{\tilde{\Delta }}_{z}^{T}{\dot{\tilde{\Delta }}}_{z}+\frac{1}{2m\sigma }\tilde{m}\dot{\tilde{m}}$$

Substituting Equations (22) and (23) into Equation (25) gives

(26) $$\begin{array}{cc} \dot{V} & =s_{z}^{T}(\frac{\hat{m}{\bar{U}}_{v}-{\hat{\Delta }}_{z}+\Delta_{z}}{m}-{\bar{U}}_{v}-c_{1}s_{z})+\frac{1}{m\eta }{\tilde{\Delta }}_{z}^{T}{\dot{\tilde{\Delta }}}_{z}+\frac{1}{m\sigma }\tilde{m}\dot{\tilde{m}}\\ & =\frac{\hat{m}{\bar{U}}_{v}s_{z}^{T}}{m}-\frac{{\hat{\Delta }}_{z}s_{z}^{T}}{m}+\frac{\Delta_{z}s_{z}^{T}}{m}-s_{z}^{T}{\bar{U}}_{v}-c_{1}s_{z}^{T}s_{z}+\frac{1}{m\eta }{\tilde{\Delta }}_{z}^{T}{\dot{\tilde{\Delta }}}_{z}+\frac{1}{m\sigma }\tilde{m}\dot{\tilde{m}}\\ & =-\frac{\tilde{m}{\bar{U}}_{v}s_{z}^{T}}{m}+\frac{{\tilde{\Delta }}_{z}s_{z}^{T}}{m}-c_{1}s_{z}^{T}s_{z}+\frac{1}{m\eta }{\tilde{\Delta }}_{z}^{T}{\dot{\tilde{\Delta }}}_{z}+\frac{1}{m\sigma }\tilde{m}\dot{\tilde{m}} \end{array}$$

Substituting ${\dot{\tilde{\Delta }}}_{z}=-{\dot{\hat{\Delta }}}_{z}$ and $\dot{\tilde{m}}=-\dot{\hat{m}}$ into Equation (26) gives

(27) $$\begin{array}{cc} \dot{V} & =-\frac{\tilde{m}{\bar{U}}_{v}s_{z}^{T}}{m}+\frac{{\tilde{\Delta }}_{z}s_{z}^{T}}{m}-c_{1}s_{z}^{T}s_{z}-\frac{1}{m\eta }{\tilde{\Delta }}_{z}^{T}{\dot{\hat{\Delta }}}_{z}-\frac{1}{m\sigma }\tilde{m}\dot{\hat{m}}\\ & =-\frac{\tilde{m}{\bar{U}}_{v}s_{z}^{T}}{m}+\frac{{\tilde{\Delta }}_{z}^{T}s_{z}}{m}-c_{1}s_{z}^{T}s_{z}-\frac{1}{m\eta }{\tilde{\Delta }}_{z}^{T}{\dot{\hat{\Delta }}}_{z}-\frac{1}{m\sigma }\tilde{m}\dot{\hat{m}}\\ & =-c_{1}s_{z}^{T}s_{z}-\frac{1}{m\eta }{\tilde{\Delta }}_{z}^{T}({\dot{\hat{\Delta }}}_{z}-\eta s_{z})-\frac{1}{m\sigma }\tilde{m}(\dot{\hat{m}}+\sigma s_{z}^{T}{\bar{U}}_{v})\\ & \leq -c_{1}s_{z}^{T}s_{z}<0 \end{array}$$

Equation (27) must hold for the update rate to be created as follows:

(28) $$\left\{\begin{array}{l} \frac{1}{m\eta }{\tilde{\Delta }}_{z}^{T}({\dot{\hat{\Delta }}}_{z}-\eta s_{z})\geq 0\\ \frac{1}{m\sigma }\tilde{m}(\dot{\hat{m}}+\sigma s_{z}^{T}{\bar{U}}_{v})\geq 0 \end{array}\right.$$

According to (28), the adaption laws are set as follows:

(29) $$\left\{\begin{array}{l} {\dot{\hat{\Delta }}}_{z}=\eta s_{z}\\ \dot{\hat{m}}=-\sigma s_{z}^{T}{\bar{U}}_{v} \end{array}\right.$$

The sliding surface $s_{z}$, the total interference estimation error ${\tilde{\Delta }}_{z}$, and the mass estimation error $\tilde{m}$ are shown to decline gradually using Equation (27). Any $s_{z}$ such that $\dot{V}\leq 0$ causes V to decrease gradually, and as a result, ${\tilde{\Delta }}_{z}$ and $\tilde{m}$ are limited. □

Equation (29) can be modified as follows to avoid the danger that an excessively large $\dot{\hat{m}}$ will result in an excessively large lift requirement.

(30) $$\dot{\hat{m}}=\mathrm{Pr}oj_{\hat{m}}(\dot{\hat{m}})=\left\{\begin{array}{l} 0,(\hat{m},m_{\max}and\dot{\hat{m}}>0)\\ 0,(\hat{m}\leq m_{\min}and\dot{\hat{m}}<0)\\ \dot{\hat{m}},\mathrm{other} \end{array}\right.$$

### 3.2. Attitude Controller Design

From Equation (14), the state space expression of the attitude subsystem is given by

(31) $$\left\{\begin{array}{c} {\dot{x}}_{7}=x_{8}\\ {\dot{x}}_{8}=a_{4}x_{10}x_{12}+b_{1}U_{2}+\Delta_{\phi }\\ {\dot{x}}_{9}=x_{10}\\ {\dot{x}}_{10}=a_{5}x_{8}x_{12}+b_{2}U_{3}+\Delta_{\theta }\\ {\dot{x}}_{11}=x_{12}\\ {\dot{x}}_{12}=a_{6}x_{8}x_{10}+b_{3}U_{4}+\Delta_{\psi } \end{array}\right.$$

According to Hypothesis 4, $\Delta_{i}(i=\phi ,\theta ,\psi )$ is bounded. Due to significant external disturbances and system model uncertainty, the inner-loop attitude control can have a substantial negative impact on the control system’s robustness and precision, which will degrade the QUAV’s flight performance. As a result, the GFTFSMC-RBF method is used in this paper’s inner-loop attitude control.

We employ the tracking differentiator (TD) to solve GFTFSMC-RBF since it requires the use of ${\dot{\phi }}_{d}$, ${\ddot{\phi }}_{d}$, ${\dot{\theta }}_{d}$, and ${\ddot{\theta }}_{d}$. TD is designed as follows [33]:

(32) $$\left\{\begin{array}{l} {\dot{x}}_{1}=x_{2}\\ {\dot{x}}_{2}=x_{3}\\ \upsilon^{3}{\dot{x}}_{3}=-2^{3/5}4(x_{1}-v(t)+(\upsilon x_{2}{)}^{9/7}{)}^{1/3}-4(\upsilon^{3}x_{3}{)}^{3/5} \end{array}\right.$$

In Equation (32), υ=0.04, v(t) is the input signal, $x_{1}$ is its trace, $x_{2}$ is an estimate of the input signal’s first order derivative, and $x_{3}$ is an estimate of the input signal’s second-order derivative.

#### 3.2.1. Global Fast Terminal Sliding Mode Controller (GFTSMC) Design

We define the attitude angle tracking error and the first- and second-order derivatives of the tracking error as follows:

(33) $$\left\{\begin{array}{c} e_{i}=x_{kd}-x_{k}\\ {\dot{e}}_{i}={\dot{x}}_{kd}-{\dot{x}}_{k}\\ {\ddot{e}}_{i}={\ddot{x}}_{kd}-{\ddot{x}}_{k} \end{array},(i=4,5,6,k=2i-1)\right.$$

Based on the GFTSMC theory [34], the sliding mode surfaces can be taken as

(34) $$s_{i}={\dot{e}}_{i}+\alpha_{i}e_{i}+\beta_{i}e_{i}{\,}^{p_{i}/q_{i}}$$

where $\alpha_{i}$ and $\beta_{i}$ are positive constants. $p_{i}$ and $q_{i}$ ($p_{i}>q_{i}$) are odd integers. The time derivative of the sliding surfaces is

(35) $${\dot{s}}_{i}={\ddot{e}}_{i}+\alpha_{i}{\dot{e}}_{i}+\beta_{i}\frac{p_{i}}{q_{i}}e_{i}{\,}^{\frac{p_{i}}{q_{i}}-1}{\dot{e}}_{i}={\ddot{x}}_{kd}-{\ddot{x}}_{k}+\alpha_{i}{\dot{e}}_{i}+\beta_{i}\frac{p_{i}}{q_{i}}e_{i}{\,}^{\frac{p_{i}}{q_{i}}-1}{\dot{e}}_{i}$$

The Lyapunov candidate functions are designed as follows:

(36) $$V_{i}=\frac{1}{2}s_{i}^{2}$$

The derivative of Lyapunov candidate functions is obtained

(37) $${\dot{V}}_{i}=s_{i}({\ddot{x}}_{kd}-{\ddot{x}}_{k}+\alpha_{i}{\dot{e}}_{i}+\beta_{i}\frac{p_{i}}{q_{i}}e_{i}{\,}^{\frac{p_{i}}{q_{i}}-1}{\dot{e}}_{i})$$

According to the system stability principle ${\dot{V}}_{i}\leq 0$, as well as according to Equation (31), the designed control rate is

(38) $$\left\{\begin{array}{c} U_{2}=\frac{1}{b_{1}}(d_{\phi }sign(s_{\phi })+\zeta_{\phi }s_{\phi }+{\ddot{x}}_{7d}-a_{4}x_{10}x_{12}+\alpha_{\phi }{\dot{e}}_{\phi }+\beta_{\phi }\frac{p_{\phi }}{q_{\phi }}e_{\phi }{\,}^{\frac{p_{\phi }}{q_{\phi }}-1}{\dot{e}}_{\phi })\\ U_{3}=\frac{1}{b_{2}}(d_{\theta }sign(s_{\theta })+\zeta_{\theta }s_{\theta }+{\ddot{x}}_{9d}-a_{5}x_{8}x_{12}+\alpha_{\theta }{\dot{e}}_{\theta }+\beta_{\theta }\frac{p_{\theta }}{q_{\theta }}e_{\phi }{\,}^{\frac{p_{\theta }}{q_{\theta }}-1}{\dot{e}}_{\theta })\\ U_{4}=\frac{1}{b_{3}}(d_{\psi }sign(s_{\psi })+\zeta_{\psi }s_{\psi }+{\ddot{x}}_{11d}-a_{6}x_{8}x_{10}+\alpha_{\psi }{\dot{e}}_{\psi }+\beta_{\psi }\frac{p_{\psi }}{q_{\psi }}e_{\psi }{\,}^{\frac{p_{\psi }}{q_{\psi }}-1}{\dot{e}}_{\psi }) \end{array}\right.$$

The index convergence rate is ${\dot{s}}_{i}=-d_{i}sign(s_{i})-\zeta_{i}s_{i}\leq 0$, where $d_{i}=\max\left|\Delta_{i}\right|+\varpi$, $\zeta_{i}>0$, and ϖ>0 are constant. This means that the designed control law ensures the stability of the system. We adjust parameters $\alpha_{i}$, $\beta_{i}$, $p_{i}$, and $q_{i}$ so that the system reaches the sliding surfaces $s_{i}=0$ in finite time $t_{s_{i}}$. By using Equation (34), we can find the following:

(39) $$t_{s_{i}}=\frac{q_{i}}{\alpha_{i}(q_{i}-p_{i})}\ln(\frac{\alpha_{i}e_{i}{\left(0\right)}^{\frac{q_{i}-p_{i}}{q_{i}}}+\beta_{i}}{\beta_{i}})$$

By choosing a sufficiently small $p_{i}/q_{i}$, the state of the system can be made to arrive at a sufficiently small neighborhood of the sliding mode surface and converge to an equilibrium state along the sliding mode surface. From Equation (38), $a_{4}x_{10}x_{12}$, $a_{5}x_{8}x_{12}$, and $a_{6}x_{8}x_{10}$ are system uncertainties.

#### 3.2.2. Global Fast Terminal Fuzzy Sliding Mode Controller Design Based on the RBF Neural Network

The RBF neural network (RBFNN) is a three-layer forward neural network with input, hidden, and output layers, where the transformation from the input layer to the hidden layer is nonlinear and the transformation from the hidden layer to the output layer is linear. Using this relationship, low-dimension input data can be converted to high-dimension input data.

In this paper, since the RBF neural network has a universal approximation property, it is used to approximate the system uncertainty. $x={\left[e_{i},{\dot{e}}_{i}\right]}^{T}$ is the input of the neural network; W is the weights of the neural networks, $h_{j}$ is the radial basis function, and it is given by

(40) $$h_{j}=\exp(\frac{{\left\|x-c_{j}\right\|}^{2}}{2b_{j}^{2}}),(j=1,2,\ldots ,5)$$

(41) $$f=W^{T}h(x)+\varepsilon$$

The output is given by

(42) $$\hat{f}={\hat{W}}^{T}H=\sum_{j=1}^{5}w_{j}h_{j}$$

where $\hat{f}$ is the system uncertainty and ε is the network tracking error. In the neural network, the neural network’s base width vector is $b_{j}$. $c_{j}=[c_{j1},c_{j2},\ldots ,c_{j5}]$ is the *j*th center vector.

In addition, the adaptive law of $\dot{\hat{W}}$ is

(43) $${\dot{\hat{W}}}_{i}=\gamma s_{i}h_{i}(i=\phi ,\theta ,\psi )$$

For the system depicted in Equation (31), the external disturbance $\Delta_{i}(i=\phi ,\theta ,\psi )$ is time-varying. If the external disturbance $\Delta_{i}$ is large, the upper bound $d_{i}$ of the disturbance must also be sufficiently large to satisfy the stability of the system; otherwise, too large a $d_{i}$ will cause the system to chatter. The fuzzy theory is better able to address this issue.

In this paper, we use a fuzzy controller to approximate external disturbances based on the universal approximation theory of fuzzy systems. First, we establish $s_{i}$ and ${\dot{s}}_{i}$ as the fuzzy controller’s inputs and $d_{i}$ as its output, and we fuzzify the input/output as follows:

(44) $$Input/Outputis\left\{\begin{array}{c} PB(Positive\;\mathrm{Big})\\ PM(Positive\;\mathrm{Middle})\\ PS(Positive\;\mathrm{Small})\\ ZO(Zero)\\ NS(Negative\;\mathrm{Small})\\ NM(Negative\;\mathrm{Middle})\\ NB(Negative\;\mathrm{Big}) \end{array}\right.$$

**Figure 3:**
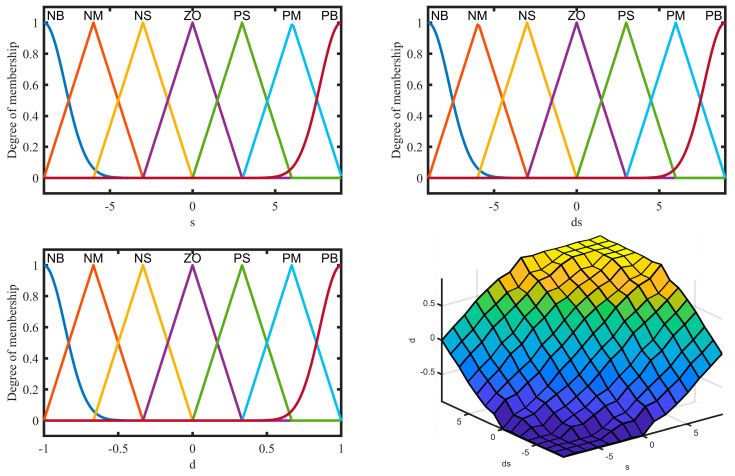
Membership functions and rule surface.

Thus, the fuzzy rules are 49. The membership function images corresponding to each input and output and the rule surface are illustrated in Figure 3.

The defuzzification method matching with the Mamdani inference mechanism is the Gravity method. It has high accuracy. The output of the fuzzy system can be expressed as

(45) $$d_{i}=\frac{\sum_{m=1}^{49}\mu_{A}(s_{i},{\dot{s}}_{i})\cdot d_{i}^{m}}{\sum_{m=1}^{49}\mu_{A}(s_{i},{\dot{s}}_{i})}$$

where $\mu_{A}(s_{i},{\dot{s}}_{i})$ is the membership function by taking both Gaussian and triangle types. $d_{i}^{m}$ indicates the *m*th rule’s crisp output.

The integral method is used to estimate ${\hat{d}}_{i}$.

(46) $${\hat{d}}_{i}=G\int_{0}^{t}d_{i}dt$$

where G is an empirically determined scale factor. Finally, Equation (38) can be rewritten as

(47) $$\left\{\begin{array}{c} U_{2}=\frac{1}{b_{1}}({\hat{d}}_{\phi }sign(s_{\phi })+\zeta_{\phi }s_{\phi }+{\ddot{x}}_{7d}-{\hat{f}}_{\phi }+\alpha_{\phi }{\dot{e}}_{\phi }+\beta_{\phi }\frac{p_{\phi }}{q_{\phi }}e_{\phi }{\,}^{\frac{p_{\phi }}{q_{\phi }}-1}{\dot{e}}_{\phi })\\ U_{3}=\frac{1}{b_{2}}({\hat{d}}_{\theta }sign(s_{\theta })+\zeta_{\theta }s_{\theta }+{\ddot{x}}_{9d}-{\hat{f}}_{\theta }+\alpha_{\theta }{\dot{e}}_{\theta }+\beta_{\theta }\frac{p_{\theta }}{q_{\theta }}e_{\phi }{\,}^{\frac{p_{\theta }}{q_{\theta }}-1}{\dot{e}}_{\theta })\\ U_{4}=\frac{1}{b_{3}}({\hat{d}}_{\psi }sign(s_{\psi })+\zeta_{\psi }s_{\psi }+{\ddot{x}}_{11d}-{\hat{f}}_{\psi }+\alpha_{\psi }{\dot{e}}_{\psi }+\beta_{\psi }\frac{p_{\psi }}{q_{\psi }}e_{\psi }{\,}^{\frac{p_{\psi }}{q_{\psi }}-1}{\dot{e}}_{\psi }) \end{array}\right.$$

where ${\hat{f}}_{\phi }$, ${\hat{f}}_{\theta }$, and ${\hat{f}}_{\psi }$ are system uncertainties. To further reduce the chattering phenomenon, the concept of the boundary layer is introduced. An improved saturation function is used to replace the traditional sign function in the switching term of the control law. The improved saturation function can be written as [35]

(48) $$sign(s)=sat(s)=\left\{\begin{array}{ll} sign(s), & \left|s\right|\geq \Theta \\ \frac{s}{\Theta^{ϒ}}, & \left|s\right|<\Theta \end{array}\right.$$

#### 3.2.3. Stability Analysis

We define the Lyapunov function as

(49) $$V_{i}=\frac{1}{2}s_{i}^{2}+\frac{1}{2\gamma }{\tilde{W}}_{i}^{T}{\tilde{W}}_{i}$$

where γ is positive constants, ${\tilde{W}}_{i}={\hat{W}}_{i}-W_{i}^{*}$, and ${\hat{W}}_{i}$ is the predicted values of $W_{i}$.

The time derivative of $s_{i}$ along Equation (31) is

(50) $$\begin{array}{cc} {\dot{s}}_{i} & ={\ddot{e}}_{i}+\alpha_{i}{\dot{e}}_{i}+\beta_{i}\frac{p_{i}}{q_{i}}e_{i}{\,}^{\frac{p_{i}}{q_{i}}-1}{\dot{e}}_{i}={\ddot{x}}_{kd}-{\ddot{x}}_{k}+\alpha_{i}{\dot{e}}_{i}+\beta_{i}\frac{p_{i}}{q_{i}}e_{i}{\,}^{\frac{p_{i}}{q_{i}}-1}{\dot{e}}_{i}\\ & =-(f-\hat{f})-d_{i}sign(s_{i})-\zeta_{i}s_{i}-\Delta_{i} \end{array}$$

Taking the derivative of Equation (49), we can obtain

(51) $$\begin{array}{cc} {\dot{V}}_{i} & =s_{i}{\dot{s}}_{i}+\frac{1}{\gamma }{\tilde{W}}_{i}^{T}{\dot{\hat{W}}}_{i}\\ & =s(-{\tilde{W}}_{i}^{T}h_{i}-d_{i}sign(s_{i})-\zeta_{i}s_{i}-\Delta_{i})+\frac{1}{\gamma }{\tilde{W}}_{i}^{T}{\dot{\hat{W}}}_{i} \end{array}$$

Substituting Equation (43) into Equation (51), we obtain

(52) $$\begin{array}{cc} {\dot{V}}_{i} & =-d_{i}\left|s_{i}\right|-\zeta_{i}s_{i}{\,}^{2}-\Delta_{i}s_{i}\leq -\varpi \left|s\right|-\zeta_{i}s_{i}{\,}^{2}\\ & \leq -\zeta_{i}s_{i}{\,}^{2}\leq 0 \end{array}$$

According to the Lyapunov stability theory, for any $s_{i}\neq 0$, there is always $\dot{V}\leq 0$, so the system is globally stable.

## 4. Simulation and Analysis

In this section, the performance of the proposed controller is verified. Therefore, the controller obtained in this paper is compared with other controllers. One compared controller, which combines the ASMC with a SMC [35], is denoted as controller SMC; the other compared controller, which combines the ASMC with a GFTSMC [36], is denoted as controller GFTSMC. The controller obtained in this paper, which combines the ASMC and a GFTFSMC-RBF, is denoted as controller GFTFSMC-RBF.

**Figure 4:**
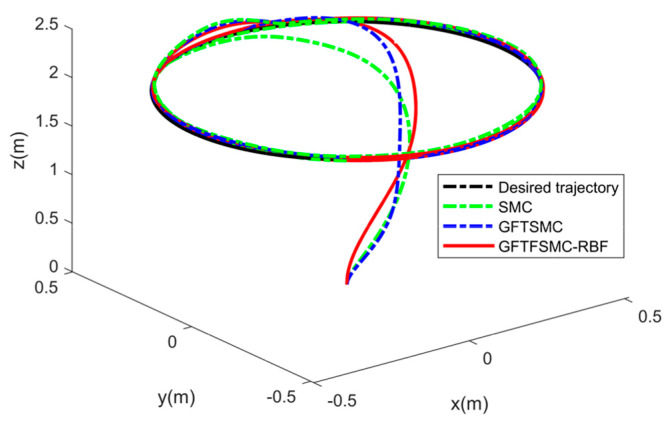
The 3D trajectory tracking of the quadrotor.

**Figure 5:**
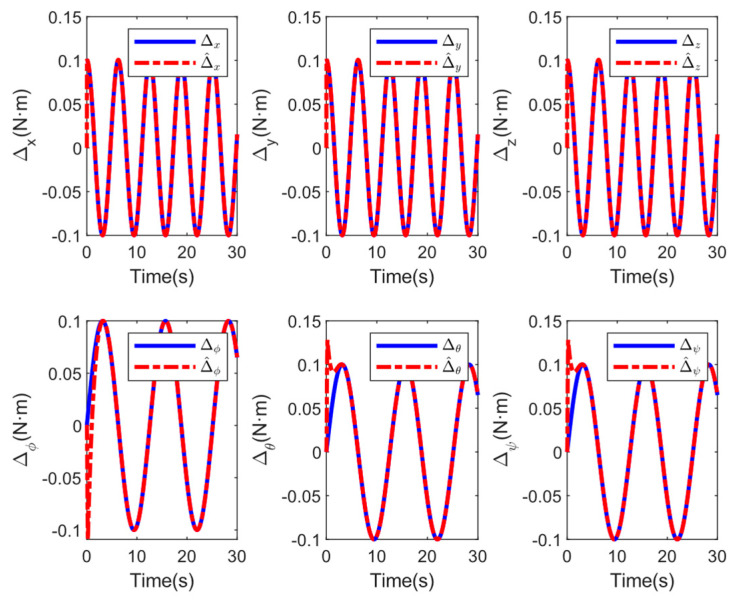
Estimations of the disturbances.

**Figure 6:**
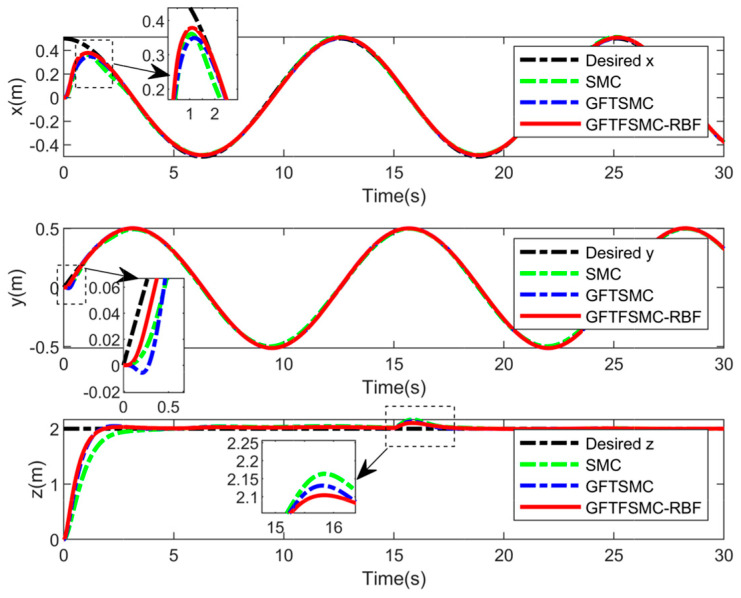
Position tracking for the quadrotor.

**Figure 7:**
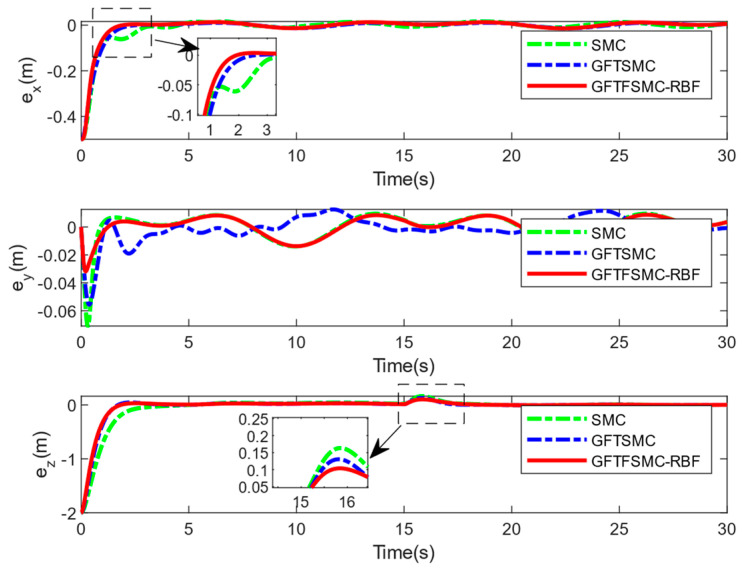
Position tracking errors.

**Figure 8:**
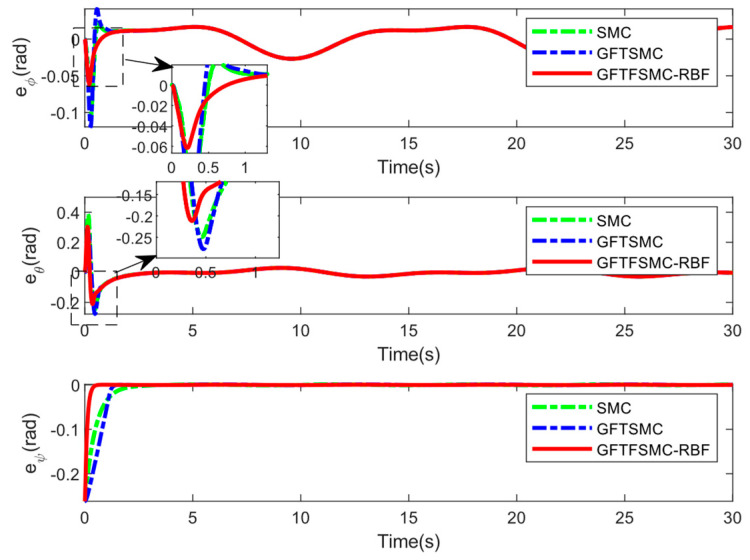
Tracking error of attitude angles.

**Figure 9:**
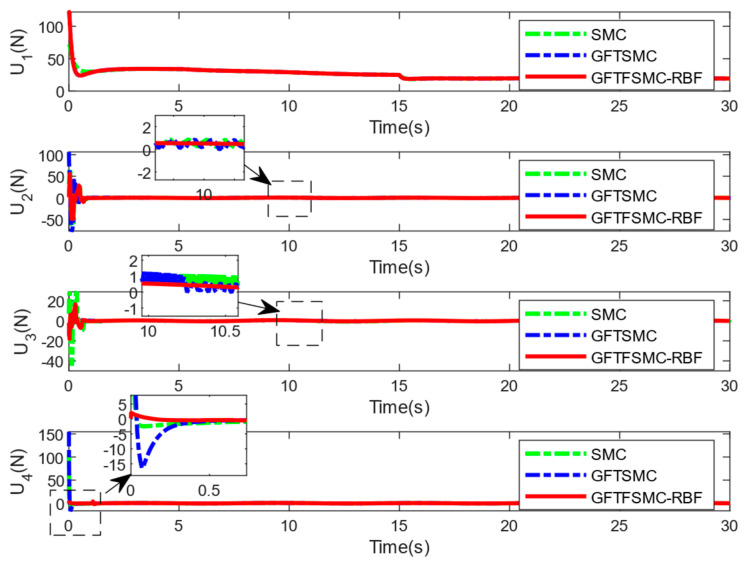
The control input of quadrotor.

**Figure 10:**
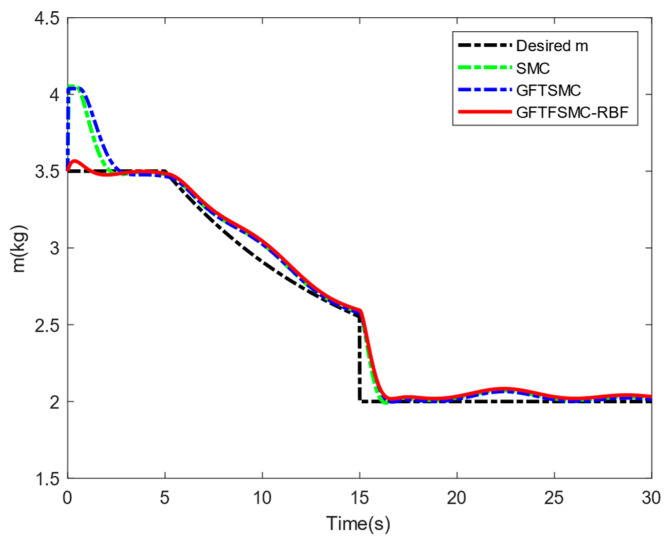
Estimation of the mass m.

Table 1 shows the QUAV parameters used in this simulation. The parameters of the controller are shown in Table 2. The compared simulations can be seen from Figure 4, Figure 5, Figure 6, Figure 7, Figure 8, Figure 9 and Figure 10 and Table 3 and Table 4.

In the simulation flight, the quadrotor tracked the 3D trajectory under external disturbances and system uncertainties. The initial state values of the quadrotor are [0, 0, 0] rad and [0, 0, 0] m. The disturbance terms are set as $\Delta_{\phi }=\Delta_{\theta }=\Delta_{\psi }=0.1\sin(t/2)$ and $\Delta_{x}=\Delta_{y}=\Delta_{z}=0.1\cos(t)$. Then, 15 s after the simulation started, the quadrotor weight suddenly changes. $\psi_{d}=\pi /12$ is the desired yaw angle. The desired trajectories are generated using the following functions:

(53) $$\left\{\begin{array}{l} x_{d}=0.5\cos(\frac{t}{2})\\ y_{d}=0.5\sin(\frac{t}{2})\\ z_{d}=2 \end{array}\right.$$

The practical change in mass is designed as follows:

(54) $$m=\left\{\begin{array}{ll} m, & 0\leq t<5\\ 1.5+2e^{-0.1(t-5)}, & 5\leq t<15\\ 2, & 15\leq t \end{array}\right.$$

Figure 4 shows the 3D flight trajectory, demonstrating that the GFTFSMC-RBF has succeeded in following the 3D flight trajectory in finite time. From Figure 5, it can be seen that the external disturbances can be accurately estimated, indicating that the control strategy proposed in this paper can improve the system’s resistance to disturbances.

From Figure 6, Figure 7 and Figure 8, the GFTFSMC-RBF obtained in this paper can achieve the fastest response speed, compared with the other two controllers. Table 3 shows the root mean square errors in x, y, and z channels obtained using the GFTFSMC-RBF; they are smaller than those obtained by the other two controllers. Thus, the tracking performance of the GFTFSMC-RBF is better than that of the other two controllers.

**Figure 11:**
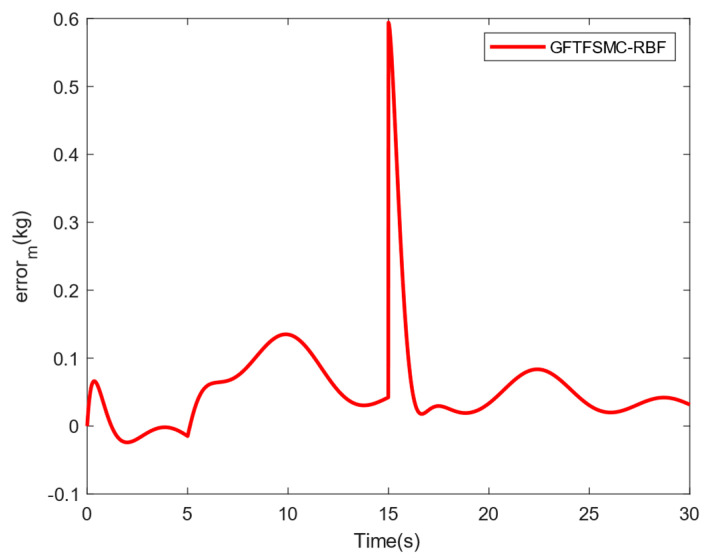
Estimation error of the mass m.

Table 4 shows the system responses in the ϕ, θ, and ψ channels. Compared with SMC and GFTSMC, the converge speeds of GFTFSMC-RBF in the three channels are all faster, obviously. Furthermore, GFTFSMC-RBF can achieve the smaller variations in the θ and ϕ angles. Observing Figure 9, it can be found that the controllers all have a chattering phenomenon, but the introduction of the radial basis function neural network and fuzzy control makes the control input and oscillation amplitude of GFTFSMC-RBF smaller than the other two controllers, which effectively suppresses the chattering phenomenon. Figure 10 and Figure 11 show that although the system is disturbed by some unknown signals, the mass can still be estimated successfully, and the estimating error is less than 0.15 kg, which is 15% of the whole mass. All in all, the controller obtained in this paper can control the time-varying mass QUAVs subjected to external disturbances and system model uncertainty, effectively. Moreover, compared with those existing control schemes, such as SMC and GFTSMC, etc., the controller with a combination of ASMC and GFTFSMC-RBF can achieve a significant improvement in flying performance.

$t_{\psi }$ is the rise time of the parameter ψ; $Z_{\max}$ and $Z_{\min}$ are the maximum and minimum responses of the parameter Z, respectively, where Z=ϕ and θ. The units of $Z_{\max}$ and $Z_{\min}$ are radian.

## 5. Conclusions

In this paper, a global fast terminal fuzzy sliding mode control scheme based on radial basis function neural network (GFTFSMC-RBF) is presented to solve the trajectory tracking problem for the time-varying mass QUAVs subjected to external disturbances and system model uncertainty. The ASMC controller has been proposed as an outer-loop controller to control the QUAV’s position. Furthermore, the GFTFSMC-RBF control has been proposed as an inner-loop controller to control the QUAV’s attitude. Finally, some simulations are given to show the robustness and efficiency of the proposed control scheme, and compared to some existing controllers, a better trajectory tracking performance is achieved for the obtained controller in this paper. Future studies will apply the proposed theoretical method to practical systems.

## Figures and Tables

**Table 1:** QUAV parameters.

Symbol	Descriptions	Value and Unit
g	Acceleration of gravity	9.81 m/s^2^
m	Total mass of the vehicle	3.5 kg
$I_{x}$	Inertial moment around x-axis	4 × 10^−3^ N·m/rad/s^2^
$I_{y}$	Inertial moment around y-axis	4 × 10^−3^ N·m/rad/s^2^
$I_{z}$	Inertial moment around z-axis	8 × 10^−3^ N·m/rad/s^2^
d	Arm length of quadrotor	0.25 m
$K_{f}$	Drag coefficient	3 × 10^−7^ N·m/rad/s
$K_{p}$	Drag coefficient	3 × 10^−5^ N·m/rad/s
$K_{ftx}$	Translation drag coefficient	5 × 10^−5^ N/m/s
$K_{fty}$	Translation drag coefficient	5 × 10^−5^ N/m/s
$K_{ftz}$	Translation drag coefficient	6 × 10^−4^ N/m/s

**Table 2:** Controller parameters of UAV.

ASMC	GFTFSMC-RBF	GFTFSMC-RBF	GFTFSMC-RBF
$k_{x}=3$	$\alpha_{\phi }=10$	$\alpha_{\theta }=10$	$\alpha_{\psi }=9.5$
$k_{y}=3$	$\beta_{\phi }=3$	$\beta_{\theta }=2.5$	$\beta_{\psi }=2.5$
$k_{z}=3$	$p_{\phi }=2$	$p_{\theta }=3$	$p_{\psi }=2$
η=0.1	$q_{\phi }=1$	$q_{\theta }=1$	$q_{\psi }=1$
σ=0.15	$\zeta_{\phi }=25$	$\zeta_{\theta }=30$	$\zeta_{\psi }=55$
ϒ=1.5	Θ=0.1	$c_{j}=[-1,-0.5,0,0.5,1]$	$b_{j}=3$

**Table 3:** Root mean square error (RMSE) of x, y, and z.

Control Methods	x	y	z
SMC	0.0606	0.0090	0.2769
GFTSMC	0.0581	0.0085	0.2346
GFTFTSMC-RBF	0.0555	0.0076	0.2229

**Table 4:** Response parameters of ϕ, θ, ψ.

Control Methods	GFTFSMC-RBF	GFTSMC	SMC
$\phi_{\max}$	0.0165	0.0411	0.0225
$\phi_{\min}$	−0.0617	−0.1195	−0.1050
$\theta_{\max}$	0.3041	0.2792	0.3801
$\theta_{\min}$	−0.2117	−0.2775	−0.2495
$t_{\psi }$	0.54	1.43	3.24

## Data Availability

Data are contained within the article.
